# TNF-α Receptor Inhibitor Alleviates Metabolic and Inflammatory Changes in a Rat Model of Ischemic Stroke

**DOI:** 10.3390/antiox10060851

**Published:** 2021-05-26

**Authors:** Shih-Yi Lin, Ya-Yu Wang, Cheng-Yi Chang, Chih-Cheng Wu, Wen-Ying Chen, Su-Lan Liao, Chun-Jung Chen

**Affiliations:** 1 Center for Geriatrics and Gerontology, Taichung Veterans General Hospital, Taichung City 407, Taiwan; sylin@vghtc.gov.tw; 2 Institute of Clinical Medicine, National Yang Ming Chiao Tung University, Taipei City 112, Taiwan; yywang@vghtc.gov.tw; 3 Department of Family Medicine, Taichung Veterans General Hospital, Taichung City 407, Taiwan; 4 Department of Surgery, Feng Yuan Hospital, Taichung City 420, Taiwan; c.y.chang.ns@gmail.com; 5 Department of Anesthesiology, Taichung Veterans General Hospital, Taichung City 407, Taiwan; chihcheng.wu@gmail.com; 6 Department of Financial Engineering, Providence University, Taichung City 433, Taiwan; 7 Department of Data Science and Big Data Analytics, Providence University, Taichung City 433, Taiwan; 8 Department of Veterinary Medicine, National Chung-Hsing University, Taichung City 402, Taiwan; wychen@dragon.nchu.edu.tw; 9 Department of Medical Research, Taichung Veterans General Hospital, Taichung City 407, Taiwan; slliao@vghtc.gov.tw; 10 Department of Medical Laboratory Science and Biotechnology, China Medical University, Taichung City 404, Taiwan

**Keywords:** hyperglycemia, insulin resistance, neuroinflammation, stroke, TNF-α

## Abstract

Hyperglycemia and inflammation, with their augmented interplay, are involved in cases of stroke with poor outcomes. Interrupting this vicious cycle thus has the potential to prevent stroke disease progression. Tumor necrosis factor-α (TNF-α) is an emerging molecule, which has inflammatory and metabolic roles. Studies have shown that TNF-α receptor inhibitor R-7050 possesses neuroprotective, antihyperglycemic, and anti-inflammatory effects. Using a rat model of permanent cerebral ischemia, pretreatment with R-7050 offered protection against poststroke neurological deficits, brain infarction, edema, oxidative stress, and caspase 3 activation. In the injured cortical tissues, R-7050 reversed the activation of TNF receptor-I (TNFRI), NF-κB, and interleukin-6 (IL-6), as well as the reduction of zonula occludens-1 (ZO-1). In the in vitro study on bEnd.3 endothelial cells, R-7050 reduced the decline of ZO-1 levels after TNF-α-exposure. R-7050 also reduced the metabolic alterations occurring after ischemic stroke, such as hyperglycemia and increased plasma corticosterone, free fatty acids, C reactive protein, and fibroblast growth factor-15 concentrations. In the gastrocnemius muscles of rats with stroke, R-7050 improved activated TNFRI/NF-κB, oxidative stress, and IL-6 pathways, as well as impaired insulin signaling. Overall, our findings highlight a feasible way to combat stroke disease based on an anti-TNF therapy that involves anti-inflammatory and metabolic mechanisms.

## 1. Introduction

Stroke is a leading cause of adult disability and mortality worldwide, particularly in the elderly population. There are two main types of stroke: ischemic stroke, resulting from the occlusion of blood vessels within the brain, accounting for >80% of all strokes; and hemorrhagic stroke, which originates from the disruption of blood vessels [1]. Despite advances in therapeutic options, surgical treatment, and tissue plasminogen activator thrombolysis, outcomes of stroke patients remain unsatisfactory [2,3]. Patients who survive acute episodes still suffer from long-term physical and/or mental sequelae [4,5]. Given the limited effective therapeutic options and the poor prognosis, a better understanding of the risk factors and pathogenesis of stroke is needed in order to prevent and reduce stroke incidence and to ease the disease burden.

Regardless of stroke types, insufficient blood perfusion causes impaired energy metabolism, leading to ATP shortages, ionic imbalance, and membrane instability. These early and intricate changes contribute to the primary brain injury. Although early recanalization is a clinical strategy for the treatment of stroke, the introduction of oxygen conversely triggers the massive production of reactive oxygen species (ROS) and cytokine overexpression, resulting in secondary brain injury and exacerbation of the disease progression [6,7]. Furthermore, inflammation and stress hyperglycemia are implicated in the pathogenesis of stroke. Stroke patients with coexisting inflammation and hyperglycemia have poor clinical outcomes [8,9,10]. In the rodent model of stroke, poststroke brain injury is accompanied by neuroinflammation and hyperglycemia. These injuries can be alleviated by means of anti-inflammatory or antihyperglycemic treatments [6,7,11,12,13]. This phenomenon in poststroke brain injury highlights the role of the interplay between inflammation and glucose metabolism and underscores the importance of inflammation and hyperglycemia as therapeutic targets for the control of stroke disease and related complications.

Biological molecules which have dual roles in inflammation and insulin resistance (in theory) are candidates for the pathogenesis and therapeutic treatment of stroke. Tumor necrosis factor-α (TNF-α) is such a probable surrogate. TNF-α is a pleiotropic molecule regulating cell proliferation, cell death, immunity, and metabolism. Through the engagement with membrane-bound TNF receptors (TNFRI and TNFRII), the post-receptor intracellular signaling of TNF-α involves c-Jun N-terminal kinase (JNK) and inhibitor of nuclear factor-κB kinase (IKK) to phosphorylate insulin receptor substrate-1 (IRS1) at the inhibitory residue serine-307 (in rodents, equivalent to serine-312 in humans), which is involved in insulin resistance [14,15]. Once injured, TNF-α can be expressed by several types of cells. TNF-α is upregulated early in peri-infarct microglia, and TNF-α-producing macrophages infiltrate the infarct and peri-infarct zone with a delay [16,17]. In stroke patients, those with high TNF-α levels [9,18] and with hyperglycema [8,9,10] develop greater neurological deficits and the outcome is worse. On the contrary, blocking TNF-α improves clinical outcomes [19]. Similar phenomena have also been observed in the stroke rodent model [20,21,22,23,24]. Importantly, posttreatment with TNF-α neutralizing antibodies or TNF-α expression inhibitors alleviates poststroke brain injury in rodents, whereas recombinant TNF-α augments it [25,26]. All evidence thus suggests that TNF-α is a potential therapeutic target for acute stroke.

Previously, we have demonstrated the development of hyperglycemia and insulin resistance in the Sprague-Dawley rat model of permanent stroke. Our findings included adipose, hepatic, and systemic inflammation and sympathetic activation [27,28,29,30]. An adequate injection of insulin protects rats from ischemic brain injury, and a non-selective β-adrenergic receptor inhibitor, propranolol, improves the action of insulin in the skeletal muscles, with a concurrent reduction in TNF-α contents [23]. To extend our previous stroke study centered on TNF-α, here we determined the effects of a TNF-α receptor antagonist, R-7050, [22] on the poststroke inflammatory and metabolic changes in a rat model of permanent stroke.

## 2. Materials and Methods

### 2.1. Cerebral Ischemia and Treatments

Male adult Sprague-Dawley rats weighing 300–330 g (*n* = 96) were purchased from BioLASCO (Taipei, Taiwan). Animal experiments were all conducted in compliance with the guidelines of the Institute, and were approved by the Animal Experimental Committee of Taichung Veterans General Hospital (IACUC approval code: La-1071584; IACUC approval date: 1 August 2018). Under the surgical procedure, rats were anesthetized with isoflurane (2–4%) and their body temperatures were controlled at 37.0 °C ± 0.5 °C. A permanent cerebral ischemia was produced by clamping both common carotid arteries, as well as the right middle cerebral artery. This procedure was the same as that described in our previously report [23]. Sham groups received similar surgical procedures but without the arterial occlusions. A single bolus of normal saline or R-7050 (5 mg/kg) was delivered intraperitoneally to the experimental or sham rats 30 min prior to surgery. All rats were euthanized for study 24 h after the completion of surgery.

### 2.2. Neurological Evaluation

The sensorimotor performance of individual rats (*n* = 8 per group) was evaluated using a modified 6-point neurological deficit scoring scheme by technicians who were blind to the treatments [23]. The scoring criteria were as follows: 0, no neurological deficit; 1, difficulty in fully extending the left forepaw; 2, unable to extend the left forepaw; 3, mild circling to the left; 4, severe cycling to the left; and 5, falling to the left.

### 2.3. Quantification of Ischemic Infarction

Rats (*n* = 8 per group) were euthanized and decapitated. Dissected brains were cut in serial coronal sections at 2-mm intervals using a Brain Slicer Matrix. After incubation in 2% triphenyltetrazolium chloride (TTC) solution at 37 °C for 30 min, areas of brain infarction were detected and measured using Image J software (National Institute of Health, Bethesda, MD, USA) [23].

### 2.4. Brain Edema

Rats (*n* = 8 per group) were euthanized and decapitated. The cortical tissues ipsilateral to the occluded middle cerebral artery were separated from the dissected brain, and dried in an oven at 110 °C for 24 h. The water content in the cortical tissue was estimated based on the wet/dry weight method [23].

### 2.5. Measurement of Oxidative Stress

Rats (*n* = 8 per group) were euthanized and decapitated. The ipsilateral cortical tissues and corresponding gastrocnemius tissues were isolated and subjected to the evaluation of lipid peroxidation using a Thiobarbituric-Acid-Reactive Substance (TBARS) assay kit (Abcam, Cambridge, UK) and glutathione (GSH) using a Glutathione Assay Kit (Cayman Chemical, Ann Arbor, MI, USA). Levels of lipid peroxidation products were expressed as malondialdehyde (MDA) equivalents.

### 2.6. Caspase 3 Activity Assay

Rats (*n* = 8 per group) were euthanized and decapitated. The ipsilateral cortical tissues separated from the dissected brains were subjected to the measurement of caspase 3 activity using a Caspase-3 Fluorometric Assay Kit (BioVision, Mountain View, CA, USA).

### 2.7. Glucose Tolerance Test

Rats (*n* = 8 per group) were deprived of a chow diet for 8 h and intraperitoneally administrated with glucose solution (2 g/kg). The Intraperitoneal Glucose Tolerance Test (IPGTT) was conducted, with glucose levels measured multiple times over a period of two hours. Blood samples were collected from the tail vein, and glucose levels were measured using a hand-held Accu-Check glucometer (Roche Diagnostics, Indianapolis, IN, USA). The total area under the curve (AUC) for IPGTT was calculated.

### 2.8. Blood Sample Analyses

Rats (*n* = 8 per group) were euthanized and then the blood was withdrawn from the left femoral artery. The obtained plasma samples were kept at −70 °C until analysis. Plasma levels of insulin (Shibayagi, Gunma, Japan), corticosterone, free fatty acids, fibroblast growth factor-15 (FGF-15), FGF-21, and C-reactive protein (CRP) (R&D Systems, Minneapolis, MN, USA) were measured using an enzyme-linked immunosorbent assay (ELISA) kit, according to the manufacturer’s instructions.

### 2.9. Measurement of Tissue Cytokines

Rats (*n* = 8 per group) were euthanized and decapitated. The ipsilateral cortical tissues and corresponding gastrocnemius tissues were isolated and subjected to the measurement of interleukin-6 (IL-6) protein contents using the ELISA kit (R&D Systems, Minneapolis, MN, USA).

### 2.10. Western Blot Analysis

Rats (*n* = 8 per group) were euthanized and decapitated. Proteins were extracted from the ipsilateral cortical tissues, corresponding gastrocnemius tissues, and bEnd.3 cell lysates using a Tissue Protein Extraction Reagent (Pierce Biotechnology, Rockford, IL, USA). Equal amounts of proteins were separated through 8% or 12% SDS-PAGE before transferring them onto PVDF membranes. Proteins of interest on the membranes were incubated with the corresponding primary antibodies, IgG-HRP, and visualized by reacting to the enhanced-chemiluminescence Western blotting reagents. The chemiluminescent blots were scanned using the G:BOX Mini multi-fluorescence and chemiluminescence imaging system (Syngene, Frederick, MD, USA). Intensities of visualized signals were quantified using Image J software (National Institute of Health, Bethesda, MD, USA). Targets of primary antibodies included the following: neuronal nuclear protein (NeuN, 1:1000), cluster of differentiation 68 (CD68, 1:1000), glial fibrillary acidic protein (GFAP, 1:1000), tumor necrosis factor-α receptor type i (TNFRI, 1:1000), IKK-α/β (1:1000), phospho-IKK-α/β (1:500), p65 (1:1000), phospho-p65 (1:500), cyclooxygenase-2 (COX-2, 1:1000), erythroid 2-related factor-2 (Nrf2, 1:1000), zonula occludens-1 (ZO-1, 1:1000), c-Jun N-terminal kinase (JNK, 1:1000), phospho-JNK (1:500), Akt (1:1000), phospho-Akt (1:500), insulin receptor substrate-1 (IRS1, 1:1000), phospho-IRS1 (Serine-307, 1:500), and glyceraldehyde 3-phosphate dehydrogenase (GAPDH, 1:3000) (Santa Cruz Biotechnology, Santa Cruz, CA, USA).

### 2.11. RNA Isolation and Quantitative Real-Time Reverse Transcriptase Polymerase Chain Reaction (RT-PCR)

Rats (*n* = 8 per group) were euthanized and decapitated. Total RNAs were extracted from the ipsilateral cortical tissues and corresponding gastrocnemius tissues using a TriZol RNA isolation reagent (Invitrogen, Carlsbad, CA, USA) and subjected to conventional cDNA synthesis and SYBR-based quantitative real-time PCR [31]. PCR was performed with the ABI StepOne^TM^ (Applied Biosystems, Foster City, CA, USA) and levels of mRNA contents were calculated using the ΔΔCT method. Oligonucleotides used for the PCR were as follows: rat IL-6, 5′-ATGAAGTTTCTCTCCGCAAGA and 5′-CTAGGTTTGCCGAGTAGACCT, and rat β-actin, 5′-AGAGGGAAATCGTGCGTGAC and 5′-CAATAGTGATGACCTGGCCGT.

### 2.12. Cell Cultures

The mouse brain endothelial cell line bEnd.3 was obtained from the Bioresource Collection and Research Center (BCRC nmuber: 60515, Hsinchu, Taiwan) and maintained at 37 °C and 5% CO_2_ in Dulbecco’s modified Eagle’s medium (DMEM), containing 10% fetal bovine serum (FBS). Cells were treated separately for 24 h with vehicle, TNF-α (50 ng/mL), R-7050 (5 μM), or in various combinations of the above.

### 2.13. Measurement of Endothelial Barrier Integrity

To measure endothelial barrier integrity, bEnd.3 cells were seeded onto Transwell inserts and allowed to grow to confluence. After experiments, the transendothelial electrical resistance (TEER) of the cell monolayer was measured with a Millicell ERS ohmmeter (Millipore, Billerica, MA, USA), with details as previously reported [31]. To measure the transendothelial permeability, dextran-FITC (0.1 μg/mL) was applied into the upper chambers for 30 min. After removing the inserts, the medium in the lower chambers was measured for fluorescence using a fluorometer (Ex 492 nm and Em 520 nm).

### 2.14. Immunofluorescence Staining

The bEnd.3 cells were seeded onto 24-well plates and allowed to grow to confluence. After experiments, cells were put in cold phosphate-buffered saline (PBS), fixed with 4% paraformaldehyde, and permeabilized with 0.1% Triton X-100. Cells were then incubated with antibodies against zonula occludens-1 (ZO-1, Invitrogen, Carlsbad, CA, USA), followed by fluorescein isothiocyanate (FITC)-conjugated secondary antibodies. Images of the stained cells were examined under a conventional epi-fluorescence microscope [31].

### 2.15. Statistical Analysis

Statistical analyses were performed using GraphPad Prism software, and data were represented as mean ± standard deviation. A two-way analysis of variance was performed to assess inter-group differences, and Dunnett or Tukey post-hoc tests were performed for comparisons. Statistical significance was set at *p* < 0.05.

## 3. Results

### 3.1. R-7050 Alleviated Postischemic Brain Injury

**Figure 1:**
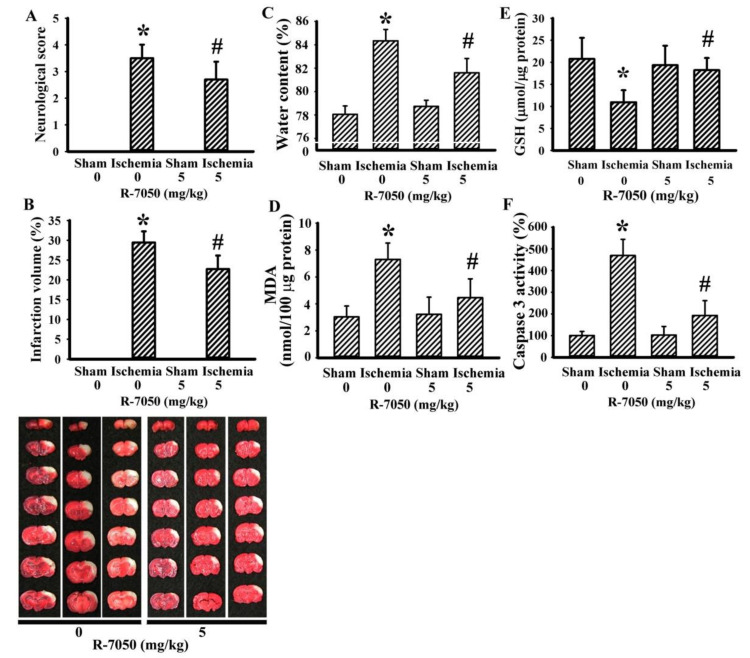
R-7050 protected against cerebral ischemia injury. Rats receiving normal saline vehicle or R-7050 (5 mg/kg) intraperitoneal injections were subjected to sham or permanent cerebral ischemia for 24 h. (**A**) Neurological deficits were evaluated by neurological score. (**B**) Representative photographs show the histological examination of brain infarction by TTC staining. The average percentage of infarction volume in the ipsilateral hemisphere is depicted. (**C**) The water contents in the ipsilateral cortical tissues were measured. (**D**) The contents of MDA in the ipsilateral cortical tissues were measured. (**E**) The contents of GSH in the ipsilateral cortical tissues were measured. (**F**) Proteins were extracted from the ipsilateral cortical tissues and subjected to an enzymatic assay of caspase 3 activity. * *p* < 0.05 vs. sham/saline and # *p* < 0.05 vs. ischemia/saline, *n* = 8.

In our experimental rats, the cerebral ischemia caused several changes, including impaired sensorimotor performance (Figure 1A), brain infarction (Figure 1B), brain edema (Figure 1C), elevation of lipid peroxidation product MDA (Figure 1D), reduction of GSH (Figure 1E), and increased caspase 3 activity (Figure 1F). All these post-ischemic changes were alleviated by R-7050 (Figure 1). Our findings indicated that R-7050 pretreatment had protective effects on ischemic brain injury.

### 3.2. R-7050 Alleviated Postischemic Inflammation

**Figure 2:**
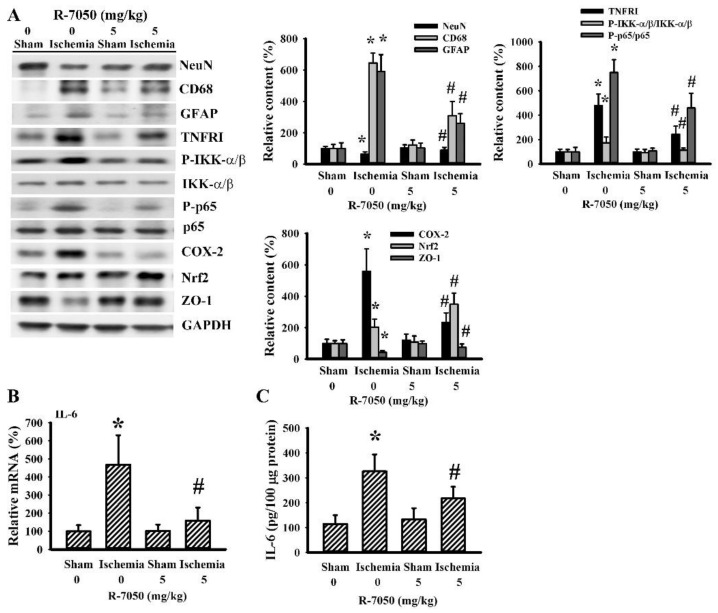
R-7050 alleviated post-ischemic brain inflammation. Rats receiving normal saline vehicle or R-7050 (5 mg/kg) intraperitoneal injections were subjected to permanent cerebral ischemia for 24 h. Proteins were extracted from the ipsilateral cortical tissues and subjected to Western blot analysis with the indicated antibodies. Representative blots and the quantitative results are shown (**A**). Total RNAs were extracted from the ipsilateral cortical tissues and subjected to quantitative real time RT-PCR for the measurement of IL-6 mRNA expression (**B**). Proteins were extracted from the ipsilateral cortical tissues and subjected to ELISA for the measurement of IL-6 (**C**). * *p* < 0.05 vs. sham/saline and # *p* < 0.05 vs. ischemia/saline, *n* = 8.

To further demonstrate R-7050’s neuroprotective effects, biochemical analyses were performed on the ipsilateral cortical tissues. Cerebral ischemia caused a reduction in neuron-related NeuN protein, with increases in both macrophage/microglia-related CD68 protein and astrocyte-related GFAP protein levels (Figure 2A). Parallel elevations were found in TNFRI protein expression, IKK-α/β protein phosphorylation, NF-κB p65 protein phosphorylation, COX-2 protein expression, Nrf2 protein expression (Figure 2A), IL-6 mRNA expression (Figure 2B), and IL-6 protein expression (Figure 2C). Conversely, there was a reduction in tight junction ZO-1 protein expression (Figure 2A). R-7050 reversed all these changes, except for that observed with Nrf2 (Figure 2). Specifically, R-7050 caused a further increase of Nrf2 protein expression (Figure 2A). These data suggest reversal effects of R-7050 on poststroke neural cell alterations, TNF-α receptor/NF-κB inflammatory signaling, IL-6 expression, and BBB disruption.

### 3.3. R-7050 Improved Postischemic Hyperglycemia

**Figure 3:**
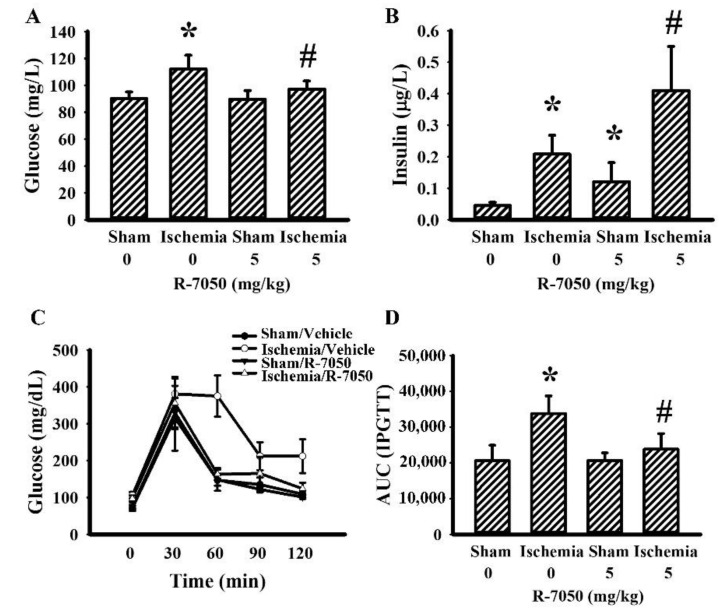
R-7050 alleviated post-ischemic hyperglycemia. Rats receiving normal saline vehicle or R-7050 (5 mg/kg) intraperitoneal injections were subjected to sham and permanent cerebral ischemia for 24 h. The blood samples were collected from 8-h fasting rats and subjected to glucose (**A**) and insulin (**B**) measurements. The 8-h fasting rats were intraperitoneally injected with a glucose solution (2 g/kg). Blood samples were collected from the tail veins at the indicated times after treatments and the levels of glucose were measured (**C**). The AUC of the glucose–time curves was calculated (**D**). * *p* < 0.05 vs. sham/saline and # *p* < 0.05 vs. ischemia/saline, *n* = 8.

**Figure 4:**
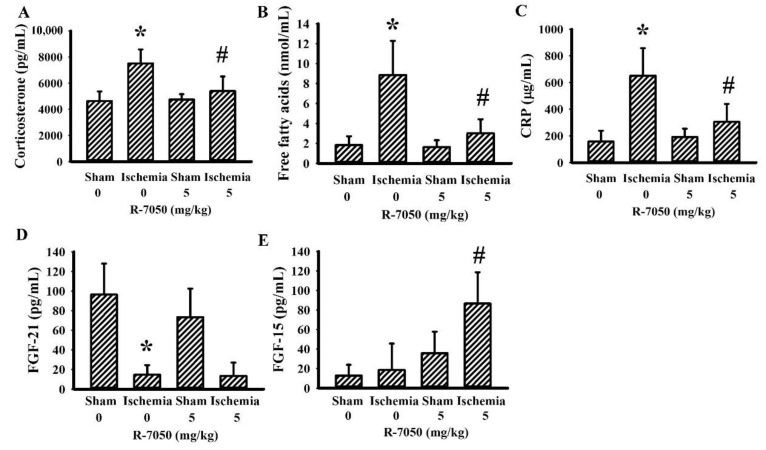
R-7050 alleviated post-ischemic changes in plasma biochemical profiles. Rats receiving normal saline vehicle or R-7050 (5 mg/kg) intraperitoneal injections were subjected to sham or permanent cerebral ischemia for 24 h. The blood samples were collected and subjected to the measurement of corticosterone (**A**), free fatty acids (**B**), CRP (**C**), FGF-21 (**D**), and FGF-15 (**E**). * *p* < 0.05 vs. sham/saline and # *p* < 0.05 vs. ischemia/saline, *n* = 8.

Biochemical parameters of glucose metabolism were determined in fasting rats. Cerebral ischemia caused rats to develop hyperglycemia (Figure 3A), hyperinsulinemia (Figure 3B), and higher post = load glucose levels after the intraperitoneal glucose injection (Figure 3C,D). R-7050 reversed hyperglycemia (Figure 3A) as well as changes in post-load glucose levels (Figure 3C,D), whereas hyperinsulinemia was augmented (Figure 3B). Upon cerebral ischemia, rats increased their circulating levels of corticosterone (Figure 4A), free fatty acids (Figure 4B), and CRP (Figure 4C), and decreased their plasma levels of FGF-21 (Figure 4D), while maintaining a constant level of FGF-15 (Figure 4E). R-7050 showed alleviative effects on corticosterone (Figure 4A), free fatty acids (Figure 4B), and CRP (Figure 4C), but it was not observed to affect FGF-21 (Figure 4D). However, R-7050 caused an augmented elevation of FGF-15 upon cerebral ischemia (Figure 4E). These findings suggest beneficial effects of R-7050 against post-ischemic hyperglycemia and impaired glucose tolerance.

### 3.4. Cerebral Ischemia’s Impairment of Insulin Action in the Gastrocnemius and the Reversal Effect of R-7050

**Figure 5:**
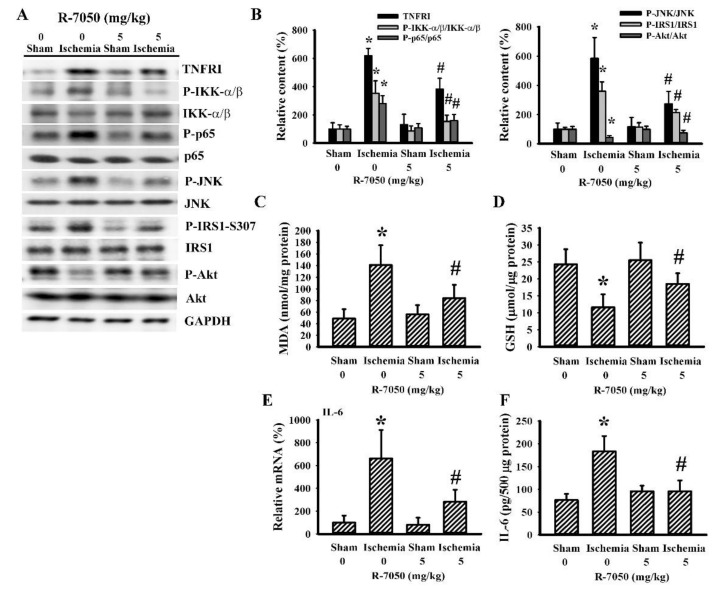
R-7050 alleviated post-ischemic gastrocnemius inflammation. Rats receiving normal saline vehicle or R-7050 (5 mg/kg) intraperitoneal injections were subjected to sham and permanent cerebral ischemia for 24 h. Proteins were extracted from the gastrocnemius muscles and subjected to Western blot analysis with the indicated antibodies. Representative blots (**A**) and the quantitative results (**B**) are shown. (**C**) The contents of MDA in the gastrocnemius muscles were measured. (**D**) The contents of GSH in the gastrocnemius muscles were measured. (**E**) Total RNAs were extracted from the gastrocnemius muscles and subjected to quantitative RT-PCR for the measurement of IL-6 mRNA expression. (**F**) Proteins were extracted from the gastrocnemius muscles and subjected to ELISA for the measurement of IL-6. * *p* < 0.05 vs. sham/saline and # *p* < 0.05 vs. ischemia/saline, *n* = 8.

Skeletal muscles are responsible for the majority of postprandial blood glucose uptake and the TNF-α inflammatory axis adversely impairs the utility of glucose, resulting in hyperglycemia and insulin resistance [14]. There was a reduction of Akt protein phosphorylation in the gastrocnemius muscles upon cerebral ischemia and the decrease was alleviated by R-7050 (Figure 5A). The reduction and reversal of Akt protein phosphorylation were paralleled by alterations in TNFRI protein expression, IKK-α/β protein phosphorylation, NF-κB p65 protein phosphorylation, JNK protein phosphorylation, IRS1 Serine-307 phosphorylation (Figure 5A,B), MDA production (Figure 5C), GSH reduction (Figure 5D), and IL-6 mRNA (Figure 5E) and protein (Figure 5F) expression. The altered parameters in the post-ischemic gastrocnemius muscles were alleviated by R-7050 (Figure 5). Therefore, the impaired insulin signaling in the gastrocnemius muscles represents a mechanism for the induction of post-ischemic hyperglycemia through TNF-α-mediated IRS1 inhibition, with R-7050 improving this impairment.

### 3.5. R-7050 Alleviated TNF-α-Induced Endothelial Barrier Disruption

**Figure 6:**
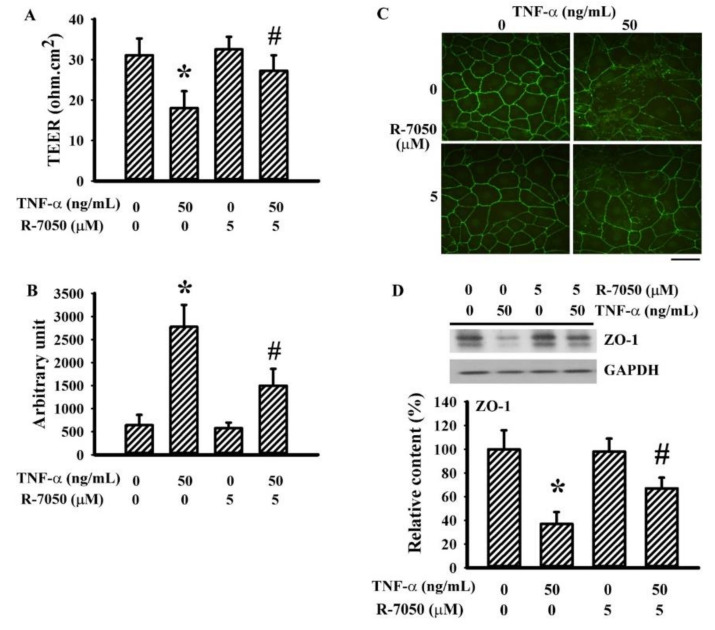
R-7050 alleviated TNF-α-increased endothelial permeability in bEnd.3 cells. Confluent bEnd.3 cells were pretreated with vehicle or R-7050 (5 μM) for 30 min before being incubated with TNF-α (0 and 50 ng/mL) for an additional 24 h. The TEER (**A**) and permeability to dextran-FITC (**B**) were measured. The cells were subjected to immunofluorescence staining with antibodies against ZO-1 (FITC) (**C**). Scale bar: 60 μm. Proteins were extracted and subjected to Western blot analysis with the indicated antibodies. Representative blots and quantitative results are shown (**D**). * *p* < 0.05 vs. untreated control and # *p* < 0.05 vs. TNF-α control, *n* = 4.

To further demonstrate the effects of R-7050 on BBB integrity, we used the bEnd.3 endothelial cell model. The integrity of the endothelial barrier was revealed by a higher TEER (Figure 6A), lower permeability to dextran-FITC (Figure 6B), ZO-1 intercellular distribution (Figure 6C), and adequate ZO-1 protein expression (Figure 6D). The sustained TNF-α exposure, as well as the resultant impaired endothelial barrier integrity and disruption, were alleviated by R-7050 (Figure 6). These findings indicate that R-7050’s preservation of BBB integrity by means of the tight junction ZO-1 protein was a likely mechanism involved in protecting the ischemic brain.

## 4. Discussion

Despite progress in stroke therapy, more efforts are needed to improve this worldwide health problem. Since hyperglycemia and inflammation coexist in stroke patients, particularly in those with worse outcomes [8,9,10], these two processes have become rational targets to combat stroke deficits and progression. Our study further demonstrated that TNF-α inhibition possessed neuroprotective, antihyperglycemic, and anti-inflammatory effects. Using a rat model of permanent cerebral ischemia, pretreatment with a TNF-α receptor antagonist, R-7050, provided protection against poststroke neurological deficits, brain infarction, edema, oxidative stress, and caspase 3 activation. In their injured cortical tissues, R-7050 had reversal effects in relation to the activation of TNFRI signaling, NF-κB inflammatory signaling, and IL-6 expression, as well as the reduction of ZO-1 protein expression. The metabolic benefits of R-7050 were accompanied by reductions in fasting glucose, glucose tolerance impairment, plasma corticosterone, plasma free fatty acids, plasma CRP, as well as an increase in plasma levels of FGF-15. Additionally, the activation of TNFRI signaling, NF-κB inflammatory signaling, oxidative stress, and IL-6 expression, as well as the impairment of insulin signaling in their gastrocnemius muscles, were all improved by R-7050. The endothelial barrier protective effects of R-7050 were further demonstrated in TNF-α-exposed bEnd.3 endothelial cells through the slowing down of the ZO-1 decline. The overall findings provided evidence highlighting a feasible way for anti-TNF-α therapy to combat stroke disease, involving anti-inflammatory and metabolic mechanisms.

Inflammation, oxidative stress, excitotoxicity, apoptosis, and ferroptosis have been implicated in the pathogenesis of neurodegenerative diseases, including stroke [32,33,34]. Among these, inflammation has a central role in stroke pathophysiology and its outcomes. The immune response starts locally, proximal to ischemic parenchyma, and subsequently expands to the ischemic penumbra, and even into systemic circulation [6,7,20,21,24,28]. The first wave of systemic inflammatory activation is followed by immunosuppression, in order to dampen inflammatory stress. This immune response causes the adaption and regeneration of neuronal cells, while the overwhelming inflammatory response conversely exacerbates poststroke brain injury [35,36,37,38,39]. Currently, several anti-inflammatory strategies, centrally or peripherally, have demonstrated protective effects on stroke-induced brain injury [23,27,35,40,41,42]. Moreover, the inhibition of brain-resident glial cells, NF-κB signaling, and peripheral immune cell central nervous system (CNS) infiltration, along with systemic depletion of circulating immune cells, also offer neuroprotective benefits [36,37]. Conversely, either excessive or inadequate depletion of immune cells exacerbates poststroke inflammation and brain injury, partly due to the deficiency of the immune defense [38,39]. In our study, neuronal injury, oxidative stress, and cell apoptosis in stroke rats were associated with brain NF-κB signaling activation and inflammatory IL-6 cytokine expression. These changes subsided with the administration of R-7050. Consistently with previous reports [22,43,44], our present study provided additional evidence of anti-inflammatory and neuroprotective effects of TNF-α inhibition in cerebral ischemia injury.

The biological activities of TNF-α are typically initiated by its engagement with TNFRs. Upon ligand engagement, TNFRs recruit and interact with downstream adaptor proteins, such as tumor necrosis factor receptor-associated factor (TRAF), TNFR-associated death domain protein (TRADD), and receptor-interacting serine/threonine protein kinase (RIP). These events guide the responses towards inflammation or other cellular activities. The mitogen-activated protein kinase (MAPK) and NF-κB axes are closely linked with TNF-α inflammatory responses [45,46]. TNF inhibitors such as the monoclonal antibody infliximab and the chimeric fusion protein etanercept can bind to TNF-α and act as decoy receptors. R-7050 can disrupt communications between TNFRs and downstream adaptor proteins. All anti-TNF-α agents show protection against stroke brain injury [20,21,22,24]. Using R-7050 as an intervention agent, the changes in enhanced brain TNFRI, NF-κB signaling molecules, and IL-6 expression in stroke rats subsided, along with increased levels of the Nrf2 protein. Nrf2 has antioxidant, anti-inflammatory, and neuroprotective effects, in addition to inhibiting TNF-α/NF-κB signaling [47,48]. In addition to disrupting the TNFR/adaptor protein complex, the current findings suggest that R-7050-enhanced Nrf2 likely represents another mechanism to alleviate inflammatory responses. At the molecular level, the tight junction ZO-1 protein is a downstream target of TNF-α signaling through proteolytic mechanisms, and its level drops in cerebral ischemia [49,50]. The preservation of the ZO-1 protein in cerebral ischemia was noted with the treatment of R-7050. Additionally, the endothelial barrier-protective effects of R-7050 via its targeting of ZO-1 protein contents were further demonstrated in TNF-α-stimulated bEnd.3 endothelial cells. Both the in vitro and in vivo findings confirmed the blockade of TNF-α inflammatory signaling and consequences by R-7050.

Impaired glucose metabolism and insulin resistance are associated with low-grade chronic inflammation, and TNF-α and IL-6 are proposed targets, inter-linking inflammation and insulin resistance [14,51]. Furthermore, higher levels of corticosterone and free fatty acids, along with several other inflammation parameters, also play a role in the development of insulin resistance [52]. R-7050 treatment improved fasting hyperglycemia and glucose intolerance, accompanied by lower plasma levels of corticosterone, free fatty acids, and CRP. Metabolic growth factors FGF-21 and FGF-15 (rodent)/FGF-19 (human) have roles in food intake, body weight control, postprandial glucose uptake, insulin sensitivity, and energy expenditure [53,54]. Despite growing evidence on the neuroprotective and anti-inflammatory effects of FGF-21 against stroke [55], our study showed that R-7050 had little effects on the poststroke FGF-21 level. Intriguingly, R-7050 caused an elevated level of FGF-15 in our stroke rats. Whether elevated FGF-15 mediates R-7050’s effects on neuroprotection, anti-inflammation, and metabolic improvement remains to be investigated.

In the regulation of glucose and lipid metabolism, in addition to from islet hormones released from the pancreas and adipose tissues, skeletal muscles have important roles in postprandial glucose uptake and storage [51]. The insulin receptor (IR)/IRS1/Akt axis is central to glucose transporter membrane shuttling, as well as glucose metabolism in skeletal muscles. Tyrosine phosphorylation is associated with the activation of IRS1 signaling, whereas serine/threonine phosphorylation has dual effects. Upon the phosphorylation of IRS1 at the inhibitory moiety serine-307 (rodent)/serine-312 (human), the insulin action is interfered with. Th evidence indicates that IKK and JNK are plausible mechanistic links between TNF-α and insulin resistance through the phosphorylation of IRS1 at the inhibitory moiety [14,15]. In our study, elevations in TNFRI, IKK-α/β phosphorylation, JNK phosphorylation, IRS1 serine-307 phosphorylation, lipid peroxidation, GSH reduction, and IL-6 expression, along with a reduction in Akt phosphorylation, were observed in the gastrocnemius muscles of stroke rats, and these alterations were alleviated by R-7050. The positive effects of R-7050 in terms of improving poststroke hyperglycemia and glucose intolerance can likely be attributed to the inhibitory effects on TNFR/IKK and TNFR/JNK signaling. However, these effects may be secondary to the suppression of IL-6, since sustained IL-6 exposure is another mechanism inducing insulin resistance in skeletal muscles [14]. Therefore, this underscores the importance of upcoming investigations centered on IL-6.

## 5. Conclusions

Hyperglycemia and inflammation are commonly linked to harmful health consequences. Inflammatory stress interferes with insulin action, resulting in hyperglycemia. High concentrations of glucose further augment inflammatory responses [14,15,56]. This vicious cycle between hyperglycemia and inflammation, as in the event of a stroke, contributes to organ damage and other metabolic disorders. Accordingly, agents or strategies that interrupt this vicious cycle, such as anti-TNF-α treatment, are promising treatment options in preventing disease progression. In this study, we have provided experimental evidence in a rat cerebral ischemia model, regarding the suppressive effects of TNF-α receptor inhibitor R-7050 on brain injury, neuroinflammation, hyperglycemia, glucose intolerance, skeletal inflammation, and insulin resistance. Based on previous reports, as well as ours [20,21,22,24], we propose that for the treatment of acute stroke, anti-TNF-α therapy shows good potential in providing anti-inflammatory and neuroprotective effects. Further investigation is needed to detail R-7050’s mechanisms of action, as well as its effects on other targets beyond TNF-α/TNFR, before these results can be translated into clinical practice.

## Data Availability

No new data were created or analyzed in this study. Data sharing is not applicable to this article.
